# Primary Leiomyosarcoma of the Kidney

**DOI:** 10.4061/2010/652398

**Published:** 2010-02-03

**Authors:** Kusuma Venkatesh, Monika Lamba Saini, S. R. Niveditha, Chaithra Krishnagiri, Sudarshan Babu

**Affiliations:** ^1^Department of Pathology, Kempegowda Institute of Medical Sciences, Bangalore 560004, India; ^2^Department of Surgery, Kempegowda Institute of Medical Sciences, Bangalore 560004, India

## Abstract

Primary leiomyosarcoma of the kidney is a rare tumor with an aggressive behaviour. A 55-year-old woman presented with a left sided abdominal mass in our outpatient department. Radiologic investigations revealed the mass to be renal in origin with colonic adhesions for which radical nephrectomy and hemicolectomy were done. The tumor completely appeared to replace the left kidney and had a whorled character focally on cut section. Microscopically, spindle cells having malignant features with cigar shaped nuclei were seen. The smooth muscle origin of the cells was confirmed by immunohistochemical positivity for smooth muscle actin. Sarcomatoid variant of the renal cell carcinoma was ruled out as the tumor was negative for cytokeratin. Tumors with spindle cell morphology in the kidney should not always be taken for a sarcomatoid variant of renal cell carcinoma and should be investigated thoroughly.

## 1. Introduction

 Renal sarcomas are rare tumors. They constitute only 1%-2% of malignant renal tumors in the adulthood [1]. Though leiomyosarcoma is the most common histologic type of the renal sarcoma (50%–60%), the information available about renal leiomyosarcomas is limited. We report a case of renal leiomyosarcoma with colonic adhesions and also review the relevant literature.

## 2. Case Report

 A 55-year-old woman presented with large palpable mass on the left side of the abdomen with dull aching pain in the flank and abdomen. Surprisingly, she had noticed the mass one month earlier on turning or bending forward. She had no haematuria, fever, or history of bowel disturbances. Previous medical history was not significant.

 On examination, vitals were within normal limits. Physical examination revealed a firm multinodular, mildly tender mass in the left hypochondrium extending up to left iliac, left lumbar, and umbilical region. The mass moved with respiration. No other organomegaly was present. Laboratory investigations revealed normocytic hypochromic anaemia with a raised ESR. Serum chemistry levels were normal. Urinalysis showed 2–5 polymorphonuclear cells per high power field without any evidence of microscopic haematuria.

 Abdominal ultrasonography showed a large heterogenous mass originating from lower half of left kidney and occupying more than three fourths of it. A portion of uninvolved left upper pole showed hydronephrotic changes. Computed tomographic scan revealed a large left renal mass without any vascular or hepatic metastasis. There was no calcification in the lesion. A provisional diagnosis of renal cell carcinoma was made. Left radical nephrectomy and left hemicolectomy revealed a renal mass adherent to descending colon. The tumor appeared to completely replace the kidney and was adherent to the colonic segments.

**Figure 1:**
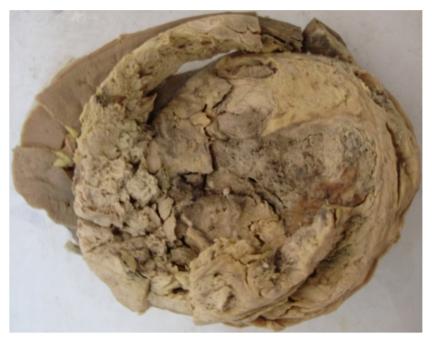
Tumor showing areas of necrosis, haemorrhage with a small portion of dilated appearing kidney.

**Figure 2:**
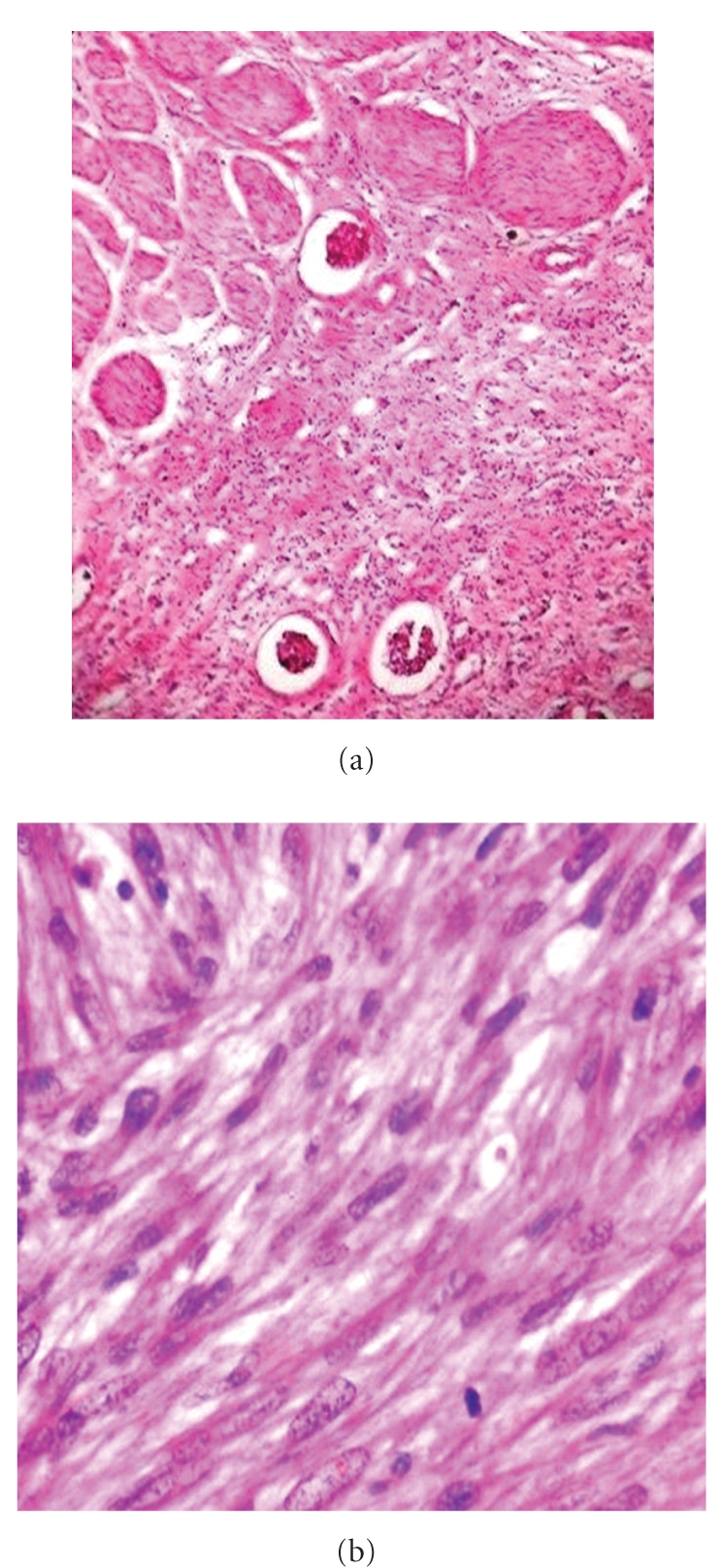
(a) Spindle cells arranged in interlacing bundles [10×]. (b) Cells with pleomorphic nuclei with blunt ends & eosinophilic cytoplasm [40×].

**Figure 3:**
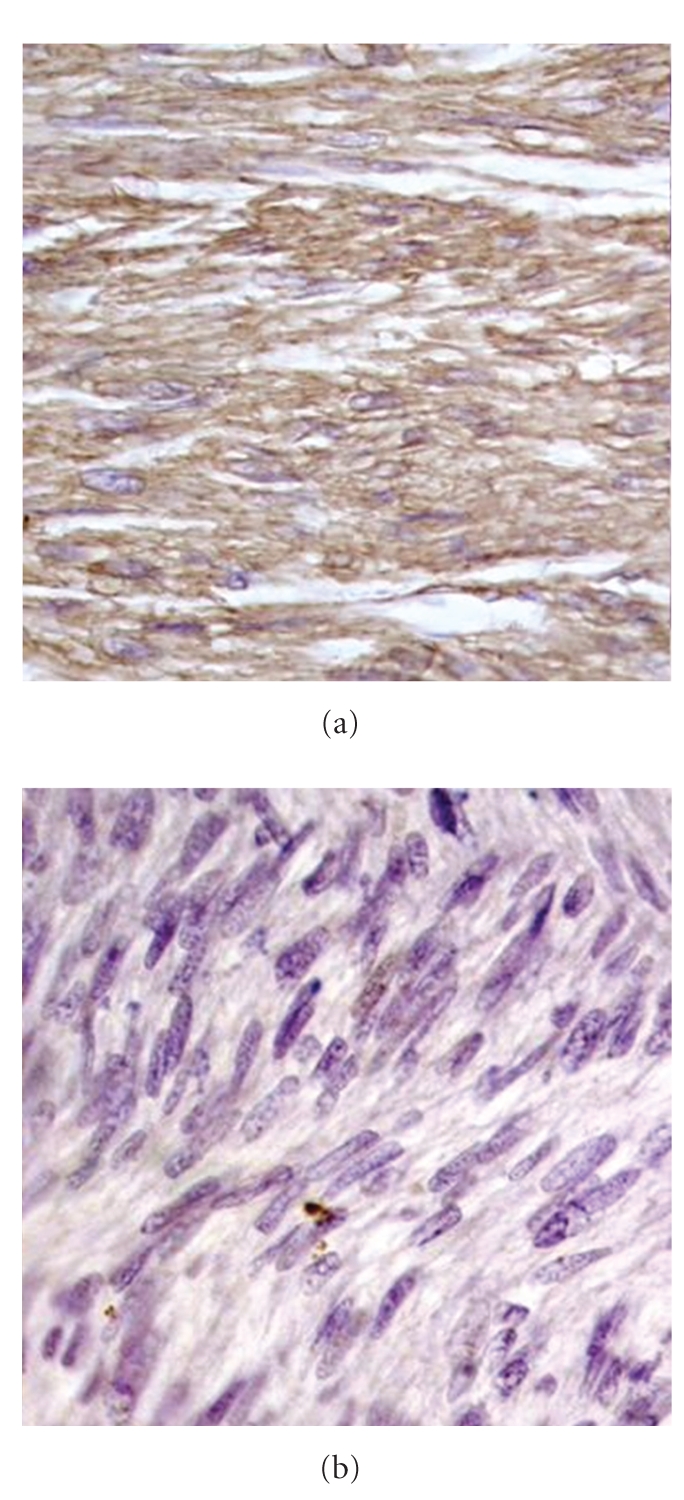
(a) Positive immunostaining for smooth muscle actin [40×]. (b) Cytokeratin negativity [40×].

 The excised mass measured 20 × 16 × 12 cms. Adherent colonic segments measured 12 cms in length. The tumor was bulky, had a nodular, lobulated external surface, and almost completely replaced the left kidney. Only a small portion of dilated appearing kidney measuring 3 × 4 cms was seen on cut section. Cut section also revealed a fleshy tan white mass with areas of necrosis, cystic degeneration, and haemorrhage [Figure 1]. The tumor was separated from the adjoining renal parenchyma by a capsule. The capsule was invaded at places by the tumor. A whorled character resembling leiomyoma was evident focally. Multiple sections studied from the mass showed a malignant tumor comprising of spindle cells arranged in interlacing bundles, whorled pattern, and sheets [Figure 2(a)]. The individual cells were spindle shaped with abundant eosinophilic cytoplasm. The nuclei were pleomorphic, elongated, and vesicular with blunt ends [Figure 2(b)]. Histologically, cells had features of smooth muscle cells. The mitotic rate was 5-6/hpf. Areas of necrosis, cystic change, and lymphoplasmacytic infiltrate were seen. Extensive sampling also failed to reveal any focus of renal cell carcinoma. Renal vessels were also free of tumor. A diagnosis of leiomyosarcoma of left kidney was made which was confirmed with positive immunostaining for smooth muscle actin [Figure 3(a)]. Desmin was also focally positive while cytokeratin was negative [Figure 3(b)]. HMB-45 and CD117 were also negative.

## 3. Discussion

 Leiomyosarcoma is a malignant tumor of smooth muscle component of soft tissue. It is essentially a tumor of adults or elderly but cases have also been reported in children [2]. Apart from the uterus, soft tissue leiomyosarcoma commonly occurs in the retroperitoneum, and also arises from the blood vessels. Leiomyosarcomas of nonperitoneal soft tissue sites usually involve the lower extremity but they can occur in the head and neck region also [3].

 Primary leiomyosarcomas are rare in the kidney and represent 1%-2% of all malignant renal tumors [1]. They appear to arise from renal capsule or smooth muscle tissue of the vessels or renal pelvic wall. The mean age at presentation is 50–60 years with a female preponderance. Grossly, the tumors look like leiomyomas with a well-circumscribed margin and whorled cut surface. The malignant counterpart, however, appears fleshy and has areas of necrosis, haemorrhage, and cystic degeneration [4]. Leiomyosarcomas rarely metastasize to the kidney. In case of metastasis, they appear as intraparenchymal lesion or as a microscopic diagnosis. Bulky tumors that replace and invade the renal tissue are typical of renal leiomyosarcoma as seen in this case.

 Microscopically, leiomyosarcomas show characteristics of smooth muscle tumor with alternating fascicles of spindle shaped cells. The cells have blunt ended, nontapering nuclei and eosinophilic cytoplasm [5]. Indicators of malignancy are necrosis, nuclear pleomorphism, and more than rare mitotic figures [6]. Grignon et al. [6] have recommended that large smooth muscle tumors should be treated with high suspicion unless proved otherwise. Focal myxoid change has also been reported [5].

Epithelioid angiomyolipoma, a variant of angiomyolipoma, can be mistaken for a leiomyosarcoma. Occasionally, the smooth muscle cells are epithelioid and exhibit nuclear atypia. They are negative for epithelial markers [7] but positive for smooth muscle and melanocytic markers. Another important differential diagnosis is sarcomatoid variant of renal cell carcinoma. Morphologically, this tumor lacks the alternating fascicles, is more pleomorphic, and usually has foci of typical renal cell carcinoma. Absence of smooth muscle markers with cytokeratin positivity is supportive of a diagnosis of carcinoma [5]. Primary monophasic synovial sarcoma of the kidney also shows monophasic spindle cells. The spindle cells are plump with irregular cell borders. They tend to grow in sheets and usually have entrapped renal tubules within them in the form of cysts. However, these tumors show positivity for Bcl-2 [8]. Fibrosarcoma and malignant peripheral nerve sheath tumor are other differential diagnoses to be considered.

The most common presenting sign is an abdominal mass with or without pain and haematuria similar to renal cell carcinoma [1]. Leiomyosarcomas, metastasizing to lungs, liver small intestine, and colon have been discussed but renal leiomyosarcomas with adherence of colonic segments only without any microscopic evidence of metastasis are rare. Sonography demonstrates multinodular masses sometimes defining the origin. Computed tomography imaging shows multinodular low-density areas with high-density septum- like structures [9].

Radical nephrectomy is the treatment of choice for renal leiomyosarcoma [1]. However, chemotherapy and radiotherapy are also recommended considering the aggressive behavior of the neoplasm. Despite resection, the tumor shows an unfavourable prognosis, metastasizing to lungs, liver and colon.

## 4. Conclusion

A nodular mass with whorled cut surface resembling leiomyoma should raise suspicion of a smooth muscle tumor in the kidney. This tumor should be sampled extensively. Histopathology alone is not sufficient to give a definite diagnosis. Immunohistochemistry to prove the smooth muscle cell origin of the tumor is essential along with exclusion of sarcomatoid type of renal cell carcinoma.

##  Conflict of Interest

All the authors wish to state that there were no conflicts of interest pertaining to the present study.

##  Funding Source

There was no funding source related to this study. All the costs pertaining to the publication of this article were borne by the corresponding author.

##  Ethical Issues

 There were no ethical issues involved as the excision; histopathology and immunohistochemical studies were done as a part of the treatment for which necessary consent was taken from the patient. Approval was also sought for publication from the patient.
